# *In silico* geometric and energetic data of all possible simple rotamers made of non-metal elements

**DOI:** 10.1016/j.dib.2020.105442

**Published:** 2020-03-19

**Authors:** Taweetham Limpanuparb, Sopanant Datta, Kridtin Chinsukserm, Peerayar Teeraniramitr

**Affiliations:** a Mahidol University International College, Mahidol University, Salaya, Nakhon Pathom 73170, Thailand; b Mahidol University International Demonstration School, Mahidol University, Salaya, Nakhon Pathom 73170, Thailand; c Faculty of Medicine Vajira Hospital, Navamindradhiraj University, Bangkok, Thailand

**Keywords:** Rotamers, Haloethanes, Relative stability, Steric effects

## Abstract

This article presents theoretical data on geometric and energetic features of halogenated rotamers of the following backbone structures, C—C, N—N, P—P, O—O, S—S, N—P, O—S, C—N, C—P, C—O, C—S, N—O, N—S, P—O and P—S. The data is considered to be comprehensive combinations of non-metal elements in the form abcx–ydef whereby a,b,c,d,e,f are halogen (fluorine to iodine), hydrogen or a lone pair and x,y are carbon, nitrogen, phosphorus, oxygen and sulfur. Data were obtained from *ab initio* geometry optimization and frequency calculations at HF, B3LYP, MP2 and CCSD levels of theory on 6-311++G(d,p) basis set. In total, 8535 non-enantiomeric structures were produced by custom-made codes in Mathematica and Q-Chem quantum chemical package. Extracted geometric and energetic data as well as raw output files, codes and scripts associated with the data production are presented in the data repository.

Specifications table

**Table:** 

Subject	Chemistry
Specific subject area	Physical and theoretical chemistry/spectroscopy
Type of data	Tables and Q-Chem output files
How data were acquired	Quantum chemical computation on Q-Chem 5.2.1, developer version
Data format	Raw and analyzed
Parameters for data collection	Hartree-Fock (HF)/6-311++G(d,p),
	Becke, 3-parameter, Lee–Yang–Parr (B3LYP)/6-311++G(d,p),
	Second order Møller–Plesset perturbation theory (MP2)/6-311++G(d,p), Coupled Cluster Singles and Doubles (CCSD)/6-311++G(d,p)
Description of data collection	Data were obtained from ab initio geometry optimization and frequency calculations. In total, 8535 non-enantiomeric structures were produced and processed by custom-made codes.
Data source location	Mahidol University, Salaya, Thailand Latitude and longitude: 13.792790, 100.325707
Data accessibility	Repository name: mendeley.com Data identification number: DOI: 10.17632/m2h6yg9nzp Direct URL to data: https://data.mendeley.com/datasets/m2h6yg9nzp

## Value of the data

• The origin of energetic preference for staggered structure in ethane [1], [2], [3], [4], [5] and gauche structure in 1,2-difluoroethane [6], [7], [8], [9], [10], [11] has long been debated and sometimes controversial. The comprehensive data set presented in this article fills in the gap in the literature and can be used for further analysis and discussion in relevant topics such as gauche effect [7,^8^,12] and bent bond [7,13].
• Similar to cis effect where the *cis* or (*Z*) isomer is more stable than *trans* or (*E*) isomer [14] and relative stability of positional isomers of substituted benzenes [15], gauche effect is demonstrated in this data set by many examples where steric hindrance alone fails to account for the observed relative stability trend.
• For reference purpose, 15665 rotamers are identified with internal numbering, SMILES and PubChem CID. (Out of 15665 rotamers, 1713 rotamers (11%) are identified with CID, of which only 631 are unique.) These can be used in future theoretical or experimental work involving two-center non-metal rotamers.
• Source codes and raw data are available for reproduction of the work and further analysis. For example, molecular dipole moment and vibrational spectrum can be extracted from the raw output. Source codes can be used to generate molecules of related classes for further calculation.

## 1 Data description

There are 15 folders for C—C, N—N, P—P, O—O, S—S, N—P, O—S, C—N, C—P, C—O, C—S, N—O, N—S, P—O and P—S. In each folder, there are four subfolders for four different methodologies, HF, B3LYP, MP2 and CCSD. In addition to raw output files (.out) and geometry in *Z*-matrix and Cartesian coordinate format (.xyz), the following summary table files (.csv) are provided in each subfolder:

• A single csv file in xyz subfolder containing geometric data of 7 bond lengths in Å, 12 bond angles and 9 torsional angles in degree (If lone pair(s) are involved, there will be less numbers of geometric parameters and ‘de’ is shown in place of a numerical value.)
• Energetic data, in separate csv files, include electronic energy (*E*_elec_) in a.u. (Hartree), thermal correction to enthalpy (*H*_corr_) in kcal mol^−1^, zero-point vibrational energy (*E*_ZPE_) in kcal mol^−1^ and entropy (*S*) in cal mol^−1^ K^−1^.

**Fig. 1:**
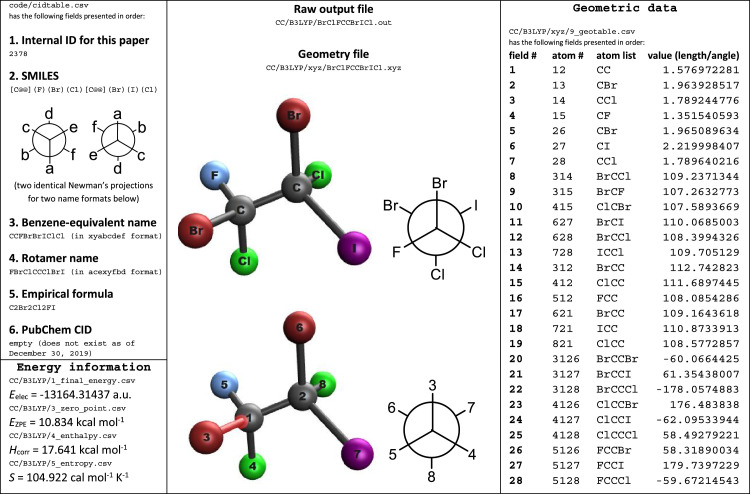
An example of data for BrClFCCBrICl rotamer calculated at B3LYP/6-311++G(d,p) level of theory.

An example of these data is shown in Fig. 1. Names for compounds exist in two different formats and due to symmetry, there are up to six ways to write these out regardless of the format. Therefore, a rotamer name may not exactly match a file name in many instances. Source codes, scripts and examples are provided in a separate folder.

## 2 Experimental design, materials, and methods

Exhaustive listing of all rotamers can be done in many different approaches. We completed our comprehensive lists of all rotamers by extending the approach we have used for substituted benzenes [15]. Rotamers as viewed by Newman projection can be equivalent to substituted benzenes with two additional conditions. First, the list of substituent elements must include a lone pair of electrons. Second, rotamers are less symmetric compared to benzenes with regards to rotation and flipping. Q-Chem 5.2.1 [16], IQmol 2.13 [17] and Wolfram Mathematica 12.0 [18] were used in the same way as described previously [15]. In addition to compounds in Table 1, Table 2, Table 3, Table 4, Table 5, Table 6, Table 7, Table 8, Preliminary calculations were also completed for all combination of single atom of C, N, P, O, S and hydrogen/halogen atoms from F to I.

**Table 1:** List of 2675 possible C—C rotamers in 210 formulas (1505 non-enantiomeric rotamers).

				Rotamers per formula		Number of rotamers	
Number of elements	Empirical formula (Distribution of elements)	Number of empirical formulas	Rotamer structure	Inclusive of enantiomers	Exclusive of enantiomers	Inclusive of enantiomers	Exclusive of enantiomers
1	C_2_α_6_ (6)	5	α_3_C−Cα_3_	1	1	5	5

2	C_2_α_5_β (1–5)	20	α_2_βC−Cα_3_	1	1	20	20
	C_2_α_2_β_4_ (2–4)	20	αβ_2_C−Cαβ_2_	3	2	60	40
			α_2_βC−Cβ_3_	1	1	20	20
	C_2_α_3_β_3_ (3–3)	10	αβ_2_C−Cα_2_β	3	2	30	20
			α_3_C−Cβ_3_	1	1	10	10

3	C_2_αβγ_4_ (1–1–4)	30	αβγC*−Cγ_3_	2	1	60	30
			αγ_2_C−Cβγ_2_	3	2	90	60
	C_2_αβ_2_γ_3_ (1–2–3)	60	αβγC*−Cβγ_2_	6	3	360	180
			αγ_2_C−Cβ_2_γ	3	2	180	120
			αβ_2_C−Cγ_3_	1	1	60	60
	C_2_α_2_β_2_γ_2_ (2–2–2)	10	αβγC*−C*αβγ (meso compound)	9	5	90	50
			αβ_2_C−Cαγ_2_ α_2_βC−Cβγ_2_ α_2_γC−Cβ_2_γ	9	6	90	60

4	C_2_αβγδ_3_ (1–1–1–3)	20	αβδC*−Cγδ_2_ αγδC*−Cβδ_2_ βγδC*−Cαδ_2_	18	9	360	180
			αβγC*−Cδ_3_	2	1	40	20
	C_2_αβγ_2_δ_2_ (1–1–2–2)	30	αγδC*−C*βγδ	12	6	360	180
			αβγC*−Cγδ_2_ αβδC*−Cγ_2_δ	12	6	360	180
			αγ_2_C−Cβδ_2_ αδ_2_C−Cβγ_2_	6	4	180	120

5	C_2_αβγδε_2_ (1–1–1–1–2)	5	αβεC*−C*γδε αγεC*−C*βδε αδεC*−C*βγε	36	18	180	90
			αβγC*−Cδε_2_ αβδC*−Cγε_2_ αγδC*−Cβε_2_ βγδC*−Cαε_2_	24	12	120	60

**Table 2:** List of 975 possible N—N (or P—P) rotamers in 70 formulas (500 non-enantiomeric rotamers).

				Rotamers per formula		Number of rotamers	
Number of elements	Empirical formula (Distribution of elements)	Number of empirical formulas	Rotamer structure	Inclusive of enantiomers	Exclusive of enantiomers	Inclusive of enantiomers	Exclusive of enantiomers
1	N_2_α_4_ (4)	5	α_2_N−Nα_2_	3	2	15	10

2	N_2_αβ_3_ (1–3)	20	αβN*−Nβ_2_	6	3	120	60
	N_2_α_2_β_2_ (2–2)	10	αβN*−N*αβ (meso compound)	9	5	90	50
			α_2_N−Nβ_2_	3	2	30	20

3	N_2_αβγ_2_ (1–1–2)	30	αγN*−N*βγ	12	6	360	180
			αβN*−Nγ_2_	6	3	180	90

4	N_2_αβγδ (1–1–1–1)	5	αβN*−N*γδ αγN*−N*βδ αδN*−N*βγ	36	18	180	90

**Table 3:** List of 45 possible O—O (or S—S) rotamers in 15 formulas (30 non-enantiomeric rotamers).

				Rotamers per formula		Number of rotamers	
Number of elements	Empirical formula (Distribution of elements)	Number of empirical formulas	Rotamer structure	Inclusive of enantiomers	Exclusive of enantiomers	Inclusive of enantiomers	Exclusive of enantiomers
1	O_2_α_2_ (2)	5	αO−Oα	3	2	15	10

2	O_2_αβ (1–1)	10	αO−Oβ	3	2	30	20

**Table 4:** List of 1875 possible N—P rotamers in 70 formulas (950 non-enantiomeric rotamers).

				Rotamers per formula		Number of rotamers	
Number of elements	Empirical formula (Distribution of elements)	Number of empirical formulas	Rotamer structure	Inclusive of enantiomers	Exclusive of enantiomers	Inclusive of enantiomers	Exclusive of enantiomers
1	NPα_4_ (4)	5	α_2_N−Pα_2_	3	2	15	10

2	NPαβ_3_ (1–3)	20	αβN*−Pβ_2_ αβP*−Nβ_2_	12	6	240	120
	NPα_2_β_2_ (2–2)	10	αβN*−P*αβ	12	6	120	60
			α_2_N−Pβ_2_ α_2_P−Nβ_2_	6	4	60	40

3	NPαβγ_2_ (1–1–2)	30	αγN*−P*βγ αγP*−N*βγ	24	12	720	360
			αβN*−Pγ_2_ αβP*−Nγ_2_	12	6	360	180

4	NPαβγδ (1–1–1–1)	5	αβN*−P*γδ $\left(\begin{array}{c} 4\\ 2 \end{array}\right)\;$= 6 structures	72	36	360	180

**Table 5:** List of 75 possible O—S rotamers in 15 formulas (50 non-enantiomeric rotamers).

				Rotamers per formula		Number of rotamers	
Number of elements	Empirical formula (Distribution of elements)	Number of empirical formulas	Rotamer structure	Inclusive of enantiomers	Exclusive of enantiomers	Inclusive of enantiomers	Exclusive of enantiomers
1	OSα_2_ (2)	5	αO−Sα	3	2	15	10

2	OSαβ (1–1)	10	αO−Sβ αS−Oβ	6	4	60	40

**Table 6:** List of 3125 possible C—N (or C—P) rotamers in 126 formulas (1625 non-enantiomeric rotamers).

				Rotamers per formula		Number of rotamers	
Number of elements	Empirical formula (Distribution of elements)	Number of empirical formulas	Rotamer structure	Inclusive of enantiomers	Exclusive of enantiomers	Inclusive of enantiomers	Exclusive of enantiomers
1	CNα_5_ (5)	5	α_3_C−Nα_2_	1	1	5	5

2	CNαβ_4_ (1–4)	20	αβ_2_C−Nβ_2_	3	2	60	40
			β_3_C−N*αβ	2	1	40	20
	CNα_2_β_3_ (2–3)	20	αβ_2_C−N*αβ	6	3	120	60
			α_2_βC−Nβ_2_	3	2	60	40
			β_3_C−Nα_2_	1	1	20	20

3	CNαβγ_3_ (1–1–3)	30	αβγC*−Nγ_2_	6	3	180	90
			αγ_2_C−N*βγ βγ_2_C−N*αγ	12	6	360	180
			γ_3_C−N*αβ	2	1	60	30
	CNαβ_2_γ_2_ (1–2–2)	30	αβγC*−N*βγ	12	6	360	180
			β_2_γC−N*αγ βγ_2_C−N*αβ	12	6	360	180
			αβ_2_C−Nγ_2_ αγ_2_C−Nβ_2_	6	4	180	120

4	CNαβγδ_2_ (1–1–1–2)	20	αβδC*−N*γδ αγδC*−N*βδ βγδC*−N*αδ	36	18	720	360
			αβγC*−Nδ_2_	6	3	120	60
			αδ_2_C−N*βγ βδ_2_C−N*αγ γδ_2_C−N*αβ	18	9	360	180

5	CNαβγδε (1–1–1–1–1)	1	αβγC*−N*δε $\left(\begin{array}{c} 5\\ 3 \end{array}\right)\;$= 10 structures	120	60	120	60

**Table 7:** List of 625 possible C—O (or C—S) rotamers in 70 formulas (375 non-enantiomeric rotamers).

				Rotamers per formula		Number of rotamers	
Number of elements	Empirical formula (Distribution of elements)	Number of empirical formulas	Rotamer structure	Inclusive of enantiomers	Exclusive of enantiomers	Inclusive of enantiomers	Exclusive of enantiomers
1	COα_4_ (4)	5	α_3_C−Oα	1	1	5	5

2	COαβ_3_ (1–3)	20	αβ_2_C−Oβ	3	2	60	40
			β_3_C−Oα	1	1	20	20
	COα_2_β_2_ (2–2)	10	αβ_2_C−Oα α_2_βC−Oβ	6	4	60	40

3	COαβγ_2_ (1–1–2)	30	αβγC*−Oγ	6	3	180	90
			αγ_2_C−Oβ βγ_2_C−Oα	6	4	180	120

4	COαβγδ (1–1–1–1)	5	αβγC*−Oδ αβδC*−Oγ αγδC*−Oβ βγδC*−Oα	24	12	120	60

**Table 8:** List of 375 possible N—O (or N—S, P—O, P—S) rotamers in 35 formulas (200 non-enantiomeric rotamers).

				Rotamers per formula		Number of rotamers	
Number of elements	Empirical formula (Distribution of elements)	Number of empirical formulas	Rotamer structure	Inclusive of enantiomers	Exclusive of enantiomers	Inclusive of enantiomers	Exclusive of enantiomers
1	NOα_3_ (3)	5	α_2_N−Oα	3	2	15	10

2	NOαβ_2_ (1–2)	20	αβN*−Oβ	6	3	120	60
			β_2_N−Oα	3	2	60	40

3	NOαβγ (1–1–1)	10	αβN*−Oγ αγN*−Oβ βγN*−Oα	18	9	180	90

Table 1, Table 2, Table 3, Table 4, Table 5, Table 6, Table 7, Table 8 provide a comprehensive listing of all rotamers considered in this work. The listing is first arranged by the number of substituent elements and pattern of empirical formulas. An explanation on how to calculate the number of chemical empirical formulas in each table is given in Table 9. Rotamer structures are also listed for each pattern. Each rotamer structure can be rotated three times unless it is symmetric (cannot rotate) or has chiral center(s) (×2 for each center). Asterisks (*) shown in Tables 1, 2, 4, Table 6, Table 7, Table 8 indicate chiral centers. There are two special cases of meso compounds in Tables 1 and 2 which have a reduced number of rotamer structures. Similar symmetrical cases were also found in previous study of substituted benzenes [15]. Since enantiomeric structures are identical in energy, only one of the two enantiomeric structures is considered for each pair. Table 10 provides an overview of all computational jobs described in this paper.

**Table 9:** Examples for number of empirical formula calculation.

Number of elements	Empirical formula (Distribution of elements)	Number of empirical formulas^a^
3	C_2_αβ_2_γ_3_ (1–2–3)	*k* = 3, *n*_1_ = 1, *n*_2_ = 1, *n*_3_ = 1 therefore $\left(\begin{array}{c} 5\\ 3 \end{array}\right)$$\frac{3!}{1!1!1!}$ = 60

3	C_2_α_2_β_2_γ_2_ (2–2–2)	*k* = 3, *n*_1_ = 3 therefore $\left(\begin{array}{c} 5\\ 3 \end{array}\right)$$\frac{3!}{3!}$ = 10

4	C_2_αβγ_2_δ_2_ (1–1–2–2)	*k* = 4, *n*_1_ = 2, *n*_2_ = 2 therefore $\left(\begin{array}{c} 5\\ 4 \end{array}\right)$$\frac{4!}{2!2!}$ = 30

5	C_2_αβγδε_2_ (1–1–1–1–2)	*k* = 5, *n*_1_ = 4, *n*_2_ = 1 therefore $\left(\begin{array}{c} 5\\ 5 \end{array}\right)$$\frac{5!}{4!1!}$ = 5

a The number of empirical formulas is calculated by using the expression (5k)$\frac{k!}{\prod n_{i}!}$, where• 5 is the number of possible substituent elements (H, F, Cl, Br, I),• *k* is the actual number of substituent elements and• ∏*n_i_*! is the product of the factorial of the number of substituent elements with the same subscript.

**Table 10:** Summary of 43450 computational jobs included in this paper (opt for geometry optimization and freq for frequency calculation).

Class of compound	Number of rotamers		HF		B3LYP		MP2		CCSD	
	All	non-enantiomeric	opt	freq	opt	freq	opt	freq	opt	freq
Preliminary CNPOS	175	170	All	All	All	All	All	–	All	–
C—C	2675	1505	All	All	All	All	All	–	23	–
N—N	975	500	All	All	All	All	All	–	30	–
P—P	975	500	All	All	All	All	All	–	30	–
O—O	45	30	All	All	All	All	All	All	All	–
S—S	45	30	All	All	All	All	All	All	All	–
C—N	3125	1625	All	All	All	All	All	–	33	–
C—P	3125	1625	All	All	All	All	All	–	33	–
C—O	625	375	All	All	All	All	All	–	33	–
C—S	625	375	All	All	All	All	All	–	33	–
N—P	1875	950	All	All	All	All	All	–	34	–
N—O	375	200	All	All	All	All	All	–	34	–
N—S	375	200	All	All	All	All	All	–	34	–
P—O	375	200	All	All	All	All	All	–	34	–
P—S	375	200	All	All	All	All	All	–	34	–
O—S	75	50	All	All	All	All	All	All	All	–
Total	15840	8535	8535	8535	8535	8535	8535	110	665	–

All optimization jobs converged in Q-Chem before geometry information were extracted. The converged results are not necessarily the same conformer as the input. For example, some anti forms are turned into gauche forms during the geometry optimization. Almost all of the converged rotamers were confirmed to be local minima by frequency calculation (16,729 out of 16,840 jobs). However, there were 111 frequency jobs with exactly one imaginary frequency ranging from 4.9i cm^−1^ to 513.1i cm^−1^. The list of files is provided in data folder. Most of them (74) are HF calculations and the rest (37) are B3LYP. Upon closer inspection, all rotamers of O—O, S—S and O—S with an imaginary frequency (55) are in an anti form. Similarly, 28 of 34 rotamers of N—N, P—P and N—P with an imaginary frequency are in an anti form from the perspective of the two lone pairs. As these observations suggest that the anti form is not stable, gauche effect is evident in these classes of compounds.

## Competing Interests

The authors declare that they have no known competing financial interests or personal relationships that could have appeared to influence the work reported in this paper.
